# A network analysis of timing and conditions present at time of death for periviable infants (22+0–23+6 weeks) admitted to neonatal intensive care after receiving survival-focused care at birth

**DOI:** 10.3389/fped.2025.1552352

**Published:** 2025-05-15

**Authors:** J. Peterson, D. M. Smith, E. D. Johnstone, K. Harvey, A. Mahaveer

**Affiliations:** ^1^ Faculty of Biology, Medicine and Health, The University of Manchester, Manchester, United Kingdom; ^2^ Perinatal Services, St Mary’s Maternity Hospital, Manchester University NHS Foundation Trust, Manchester, United Kingdom; ^3^ North West Neonatal Operational Delivery Network, Alder Hey NHS Foundation Trust, Liverpool, United Kingdom

**Keywords:** neonates, extreme preterm, periviable, mortality, palliative care

## Abstract

**Introduction:**

Advances in neonatal care have resulted in improved survival rates for periviable infants (22 + 0–23 + 6 weeks) with increasing numbers being admitted to neonatal intensive care units across the United Kingdom. Qualitative research evidences the conflict perinatal professionals experience traversing the line between providing life-sustaining treatment to these infants, whilst not wanting to inflict a prolonged period of suffering to infants who will ultimately die. Professionals currently lack adequate prognostic tools to accurately predict pre-birth which infants will survive.

**Methods:**

This study utilises an anonymised dataset from the North West Neonatal Network to delineate time of death profiles for periviable infants admitted to neonatal intensive care units (NICU) and explores the demographics, timing and diagnoses recorded at the time of the death.

**Results:**

The data show that most periviable infants who died following admission to NICU died within the first seven days after birth [24 infants born at 22 weeks (65%) and 55 infants born at 23 weeks (52%)]. For infants born at 22 weeks who subsequently died on NICU, 89% had died within 14 days after birth. Reorientation of care was recorded as a relevant factor at the time of death in a minority of patients [23 infants (16%)].

**Discussion:**

Where active, survival-focused care has been initiated, the response of the infant to intensive care and the likelihood of their survival emerges over a relatively short timeframe after admission. This lends support to a trial of therapy approach for suitable periviable infants balancing the need to avoid iatrogenic harm to infants who will ultimately die despite intensive care, whilst not denying them the chance at survival. Management of periviable deliveries requires coordinated parallel planning and a high-quality palliative care approach throughout.

## 1 Introduction

In 2019 the British Association of Perinatal Medicine released an updated framework for the management of extremely preterm infants (1). This framework quoted survival rates in the region of 30% for infants born at 22 weeks who had been born alive (1). This updated national guidance calls for an individualised, risk-based approach to decision-making around appropriateness of instigating active, survival-focused care for periviable infants (22 + 0–23 + 6 weeks), rather than focusing on gestational age alone. In 2023, Smith et al. published a subsequent evaluation of national data demonstrating a three-fold increase in the number of periviable infants receiving survival-focused care (2). With this increase in numbers of periviable infants receiving survival-focused care, perinatal professionals and parents face the dilemma of balancing the increased chance of survival against the burden of the treatment itself.

Provision of intensive care comes with an intrinsic burden to the patient as the intensity of the treatment required is often accompanied by invasive, painful interventions and procedures. Intensive care can be a brutal environment for patients, families and healthcare professionals alike (3). The inherent burden of intensive care can be justified where there is a reasonable chance of recovery. However, in circumstances where the outcome is uncertain, or a favourable outcome is judged to be unlikely, it can be become untenable for patients and professionals to continue provision of intensive care and a redirection to comfort (or end of life) care may be appropriate (4). Making the judgement about where the balance between the harms and benefits of treatment options is particularly difficult when making the decision on behalf of another, as is the case for neonatal patients (5). Together, clinicians and parents should aim to balance the risks and benefits of various treatment and management options as they relate to that individual infant, with the clinician providing evidence-based medical information for assimilation and the parents bringing their family context, values and moral code through which this information should be considered (6). The infant is part of their individual family and was intended to be raised within that familial context. Therefore, considered and informed decisions made by the parents should be honoured and enacted by the clinical team (except in the rare circumstance of objective evidence of parental malicious intent).

Birth at periviability confers an inherently uncertain outcome. The periviable period is, by definition, the timeframe where the infant may be able to survive outside the womb contingent on a prolonged period of intensive care being provided, but even with this, the likelihood of survival is limited. For the United Kingdom in 2024, the period of periviability is considered 22 + 0–23 + 6 weeks gestation (1). For births within this periviable period parents and professionals need to determine the appropriateness of intensive care provision for these infants, or whether, in that infant's individual circumstances and risk profile, the burden of intensive care is too high and comfort care would be a more ethically justifiable option. Shared decision-making in this context carries significant practical, emotional, and ethical complexities for parents and professionals. Implementing intensive, survival-focused care for infants with risk factors placing them at extremely high risk of death may lead treating professionals to experience moral distress and professional guilt (7). Professionals may struggle to navigate the contradictory demands of attempting to balance upholding the baby's right to life, whilst also upholding their right to not be subjected to an undue burden of treatment and simultaneously to be an advocate for the parents and their right to make decisions for their baby. In situations with highly uncertain prognoses, healthcare professionals may have to enact treatment interventions selected by parents which they personally do not consider to be optimal course of management for that infant (8). Whilst decisions made by parents that reasonably fall within the ‘zone of parental discretion’ should be adhered to, the treating professional may be the person consequently having to subject the patient to the potentially painful or uncomfortable procedures and interventions needed for that treatment plan (9, 10). This could be perceived by the professional as acting against one of the central pillars of medical ethics—“Do No Harm” (11). This discordance between one's required professional actions and one's own moral code can lead to a burden of moral distress for the professional. Moral distress has been linked with increased anxiety, depression and burnout (7, 12).

The continuation of intensive care in the face of futility, or non-response, has been reported by healthcare professionals as contributing to their moral distress (13). In a survey conducted by the authors’ research group in 2022 (publication pending), professionals across perinatal specialities reported emotional and moral distress when discussing management and outcomes with parents facing periviable birth. One of the reported factors in this distress burden was the uncertainty in outcome and uncertainty about what course of action was “right” for the baby with professionals wanting to provide hope whilst avoiding doing harm. Perinatal professionals acknowledged the uncertainty inherent in assessing risk profiles for periviable infants pre-birth and that assessment of the infant after birth was helpful in refining risk assessment for mortality and morbidity and guiding subsequent management. Additionally, research shows that for parents with children with life-limiting conditions, including extreme prematurity, who have experience in making medical decisions on behalf of their children, parents often felt they did not have sufficient time to process the relevant information and make an informed decision (14). These factors present a dilemma for parents and professionals to navigate; time is required for information processing and consideration of options and, additionally, may enable the professional to make a more detailed assessment of the infant and their response to birth and stabilisation measures after birth. However, this carries the risk of the baby undergoing potentially invasive management which may ultimately be unsuccessful and the multiple distress burdens this confers to the infant, parents, and professionals.

Our previous network analysis of periviable optimisation and outcomes (published 2024) demonstrated that perinatal professionals currently lack adequate prognostic tools to accurately predict pre-birth which periviable infants will survive (15). The data from that study also indicated that the minority of periviable infants (20%) who had been admitted to NICU for active, survival-focused care survived (15). Qualitative research evidences the conflict that perinatal professionals experience traversing the line between providing life-sustaining treatment to these infants, whilst not wanting to inflict a prolonged period of suffering to infants who will ultimately die.

The aim with our current study is to explore the timing of death for those periviable infants who died on NICU despite receiving survival-focused intensive care. Perinatal professionals have to balance the need to avoid iatrogenic harm to infants who will ultimately die despite intensive care, whilst not denying them the chance at survival. Our objective is to utilise quantitative data to delineate when death occurs for this subgroup of periviable infants (those admitted to NICU for survival-focused care) and in doing so, contribute to the ethically complex discussion around the appropriateness of survival-focused care for periviable infants. To that end, this study evaluates the timing of death and conditions present at the time of death for periviable infants (22 + 0–23 + 6 weeks) born within the North West Neonatal Network over the last 5 years (2018–2022) across the change in national guidance in late 2019.

## 2 Methods

### 2.1 Data collection

Data were gathered through the North West Neonatal Operational Delivery Network [NWNODN (16)] which is made up from 22 neonatal units across the region, with two Special Care Units (SCBU; Level 1), twelve Local Neonatal Units (LNU; Level 2) and seven Neonatal Intensive Care Units (NICU; Level 3) and an additional surgical unit within a tertiary children's hospital (17). All data collected were anonymised. Data analysts from the NWNODN were able to collate data from the electronic patient record Badger.Net (18) for all periviable deliveries (22 + 0–23 + 6 weeks) across the NW network between 01/01/2018 to 31/12/2022. The study was reviewed and granted approval by the University of Manchester Psychology and Mental Health Divisional Review panel (2023-17791-31615).

The extracted data fields were gestational age, birth weight, biological sex, birth order, delivery mode, date of birth, time of birth, resuscitation interventions, admission temperature, blood sugar on admission, antenatal steroid provision (complete (2× doses), partial (1× dose), none, not recorded), magnesium sulphate provision (yes/no), date of death, time of death, cause of death and diagnoses at discharge.

### 2.2 Inclusion criteria

• Gestational age at birth 22 + 0–23 + 6 weeks (singletons and multiples were included)
• Episode one on Badger.Net occurring within the North West Network hospitals
• Alive at admission to the neonatal unit

### 2.3 Exclusion criteria

• Gestational age at birth **≤**21 + 6 weeks or **≥**24 + 0 weeks
• Infants born outside the North West Network. This exclusion was applied to infants repatriated back into the North West Network
• Intrapartum death or death in the delivery room

### 2.4 Data analysis

Descriptive statistics (percentages, means and range values) were analysed overall and stratified by gestational age in weeks. One-way ANOVA analysis was performed for comparisons across multiple groups such as birth weight and gestational age variation between time of death cohorts. Median survival time was calculated for each cohort (22 + 0–22 + 6 and 23 + 0–23 + 6 weeks). A Kaplan Meier survival curve was used to present risk of death over time and a Mantel-Cox analysis was performed for comparison of the mortality curves between the two gestational age cohorts (22 + 0–22 + 6 and 23 + 0–23 + 6 weeks). Statistical significance was set at *p* < 0.05. The data were analysed using GraphPad Prism (version 10.2.3) software.

## 3 Results

There were 143 periviable infants who died following admission to neonatal units across the North West between 2018 and 2022 (Table 1). Of these 143 infants, 37 were born at 22 + 0–22 + 6 weeks and 106 born at 23 + 0–23 + 6 weeks gestation. There was an equal split between male and female infants [69 female (48%) and 74 male (52%)]. Most infants who died had been a singleton pregnancy (116 infants), with 27 infants having been part of a multiple birth (twins or triplets; there were no higher order births recorded in this cohort). The majority had received antenatal optimisation interventions such as one or more doses of antenatal steroid (114 infants (80%) and magnesium sulphate provision [103 infants (72%)] and 80% of infants were delivered via spontaneous vaginal birth (115 infants). Birth weight varied by gestational age with a higher mean birth weight for infants in the 23-week cohort compared to 22-week cohort (568 grams compared to 490 grams respectively). There was more variation in birth weight within the 23-week cohort [314–770 grams (IQR 118 grams)] compared to the 22-week cohort (360–640 grams (IQR 80 grams). The smallest birth weight occurred in the 23-week cohort (314 grams). Both cohorts achieved target admission temperature and blood sugars on average (Table 1). However, there was large variation for both admission temperature and blood sugar for both cohorts resulting in infants in both groups being admitted hypo- and hyperthermic and with hypo- and hyperglycaemia. These factors are known to be associated with increased mortality risks in premature infants (19).

**Table 1:** Admission demographics for periviable infants who died whilst receiving intensive care by gestational age (above) and by timing of death (below).

Gestational age at birth					
		22 + 0–22 + 6		23 + 0–23 + 6	
Total infants		37	%	106	%
Antenatal magnesium sulphate	Yes	25	68	78	74
	No	12	32	28	26
	Blank	0	0	0	0
Antenatal steroids	Complete	13	35	55	52
	Partial	13	35	33	31
	Nil	0	0	0	0
	Blank	11	30	18	17
Birth weight (g)	Mean	490		568	
	Min	360		314	
	Max	640		770	
	Q1	450		512	
	Q3	530		630	
	IQR	80		118	
Admission temperature (celcius)	Mean	36.7		36.6	
	Min	32.7		34.0	
	Max	39.8		38.7	
	Q1	36.3		36.1	
	Q3	37.1		37.2	
	IQR	0.8		1.1	
Admission blood sugar (mmol/L)	Mean	4.5		3.5	
	Min	1.5		0.0	
	Max	15.3		8.4	
	Q1	2.9		2.3	
	Q3	4.8		4.3	
	IQR	2.0		2.1	
			**Age at death**		**Age at death**
Birth location	NICU	33	2 h–70 days	79	2 h–209 days
	LNU	4	2 h–5 days	18	4 h–75 days
	SCBU	0		1	8 h
	Out of hospital	0		8	2 h–74 days


Demographics by timing of death					
Birth weight (g)	**Day 0–2**	**Day 3–7**	**Day 8–14**		
Mean	548	541	547		
Min	360	371	450		
Max	770	750	720		
Q1	502	460	481		
Q3	589	616	619		
IQR	87	156	138		
One-way ANOVA	**Sum of squares**	**df**	**Mean square**	**F**	**Sig (*p* value)**
Between-treatments	1,004.35	2.00	502.18	0.06	0.94
Within-treatments	828,025.61	102.00	8,117.90		
Total	829,029.96	104.00			
Gestational age at birth	**Day 0–2**	**Day 3–7**	**Day 8–14**		
Mean	23.0	23.0	23.0		
Min	22.0	22.0	22.3		
Max	23.6	23.6	23.6		
Q1	22.6	22.6	22.6		
Q3	23.4	23.4	23.3		
IQR	0.8	0.8	0.7		
One-way ANOVA	**Sum of squares**	**df**	**Mean square**	**F**	**Sig (*p* value)**
Between-treatments	0.05	2.00	0.02	0.10	0.90
Within-treatments	23.35	102.00	0.23		
Total	23.40	104.00			
Antenatal steroids (relative % of infants within each subgroup)	**Day 0–2**	**Day 3–7**	**Day 8–14**		
Total infants	42	37	26		
Complete	21	59	50		
Partial	48	30	27		
Not recorded	31	11	23		

Min, minimum; Max, maximum; Q1, quartile 1; Q3, quartile 3; IQR, interquartile range; NICU, neonatal intensive care unit; LNU, local neonatal unit; SCBU, special care baby unit.

There were no significant differences in birth weight (ANOVA F 0.06, *p* value 0.94) or gestational age (ANOVA F 0.10, *p* value 0.90) for those infants who died across the first 14 days of life. For infants who died in the first 48 hours after birth, there were lower rates of complete antenatal steroid provision (21%) compared to infants who died later where rates of complete ANS administration were 59% for infants who died day 3%–7% and 50% for infants who died between day 8–14 after birth. Interpretation of this data is complicated by high rates of missing data (ANS provision was not recorded in 31% of infants who died in the first 48 hours of life).

### 3.1 Timing of death

**Figure 1:**
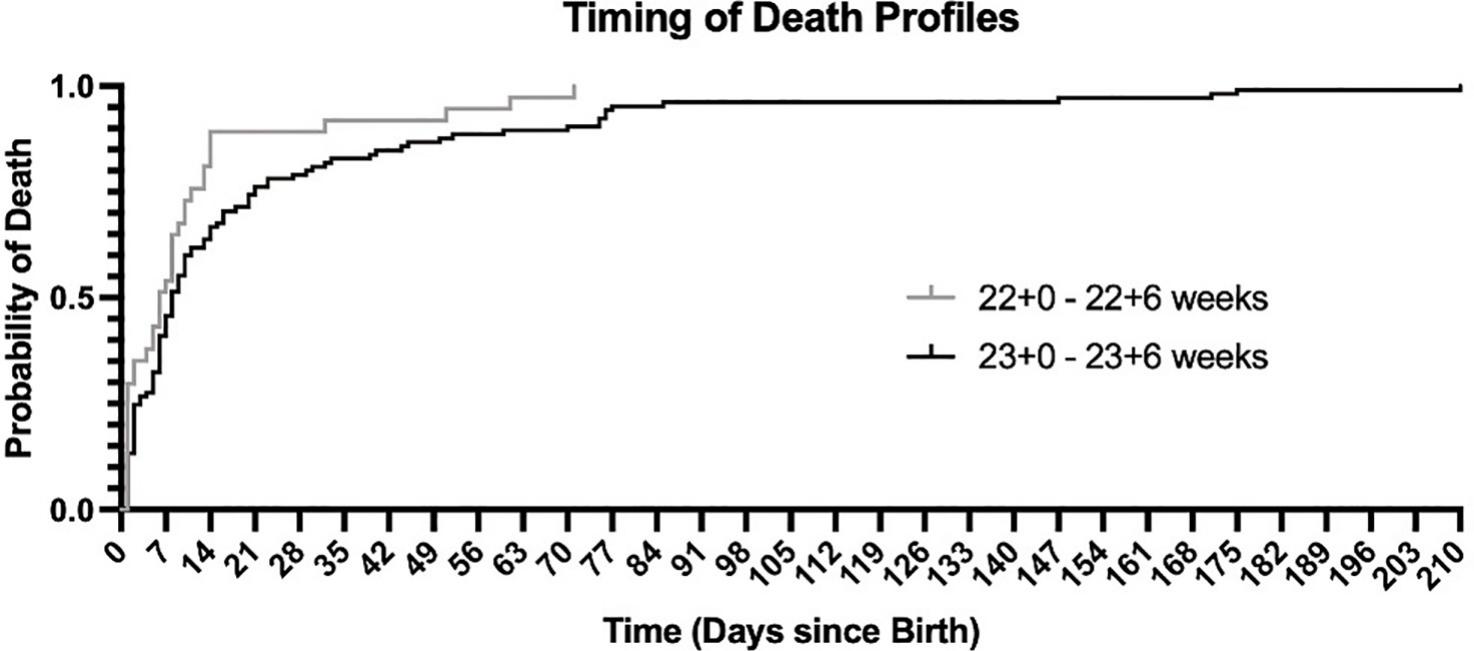
Kaplan Meier curve for periviable infants who died whilst receiving neonatal intensive care.

Timing of death was analysed by gestational age cohort (22 + 0–22 + 6 weeks and 23 + 0–23 + 6 weeks) and the profiles across these two cohorts showed differences over time. The median time to death was day 6 of life for 22 week cohort (0–70 days of life; IQR = 10) and day 8 of life for the 23 week cohort (0–209; IQR 18.5 days). Overall, the data demonstrate that for periviable infants who did not survive following admission to NICU, most infants died within the first 14 days of life. The probability of death by day 14 was 0.89 for 22 week cohort and 0.65 for the 23 week cohort (Figure 1).

For both the 22 and 23 week cohorts, the highest risk period for death was the first 72 hours after birth (0.35 and 0.28 respectively). Focusing on the 22 week cohort who died, the Kaplan Meier shows a cumulative risk of death of 0.65 within the first 7 days, and 0.89 within 14 days of birth. This profile differed significantly from the 23 week cohort, where cumulative risk of death was 0.68 by day 14 of life, after which followed a more static risk of death over subsequent weeks (between day 8–100 of life) (Figure 1; *p* value <0.005).

### 3.2 Conditions present at the time of death

**Figure 2:**
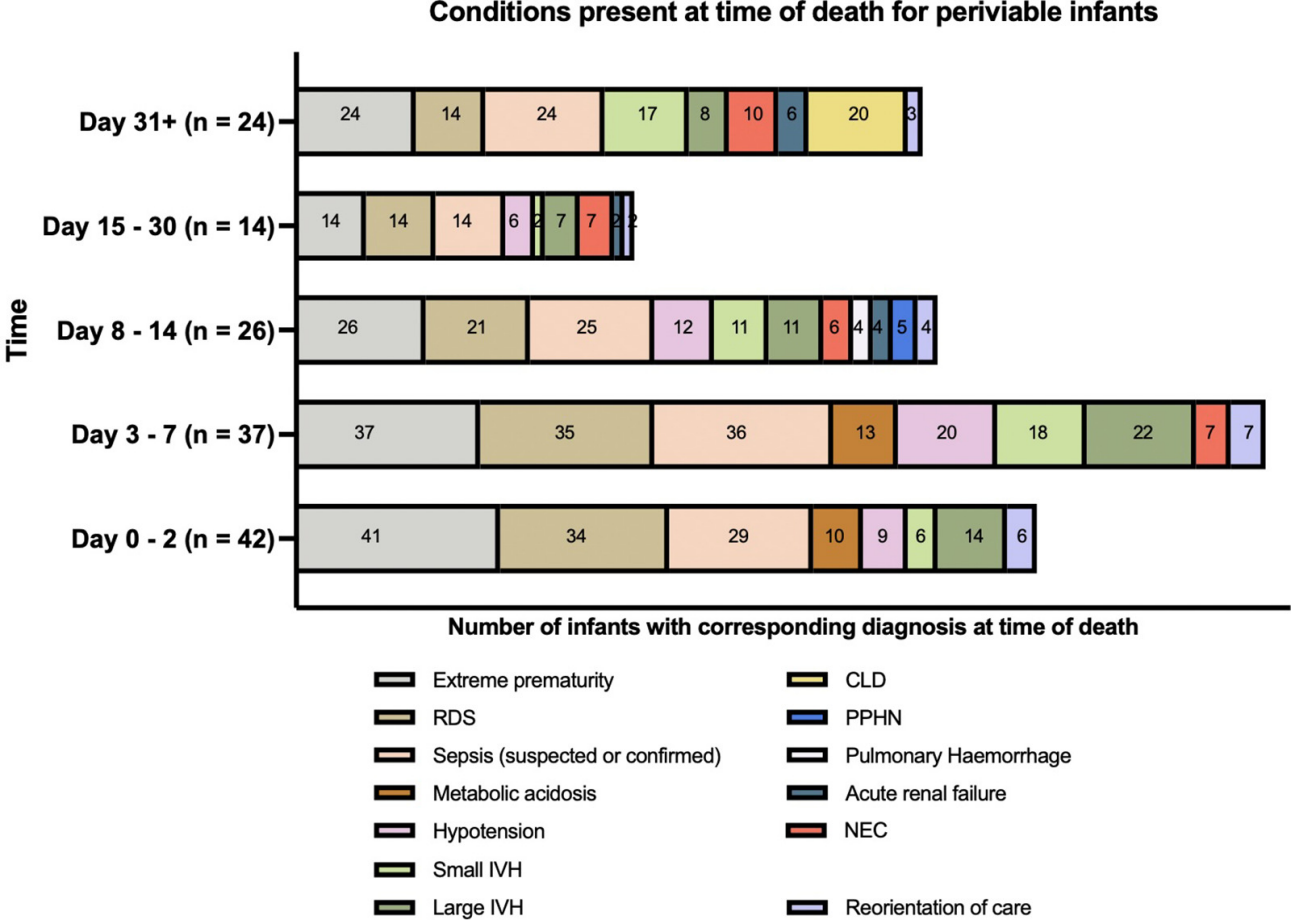
Conditions present at the time of death for periviable infants who died whilst receiving intensive care.

The profile of conditions present at the time of death varied dependent on the age of the infant (Figure 2). For periviable infants who died in the first 7 days after birth the most common conditions present at the time of death included extreme prematurity, respiratory distress syndrome, sepsis, metabolic acidosis and large intraventricular haemorrhage (grade 3–4). For infants dying between day 8–30 after birth, these earlier diagnoses remained alongside additional conditions such as necrotising enterocolitis (NEC), pulmonary haemorrhage and acute renal failure. For periviable infants who died after 30 days from birth, chronic lung disease, sepsis, acute renal failure and NEC were most frequently present at time of death.

### 3.3 Reorientation of care

From the 143 infants in this study who were periviable infants who died after admission to NICU, there were 23 infants where reorientation of care was recorded as a factor in their death (16%) (Figure 2). There was an equal split between male and female infants in this subgroup (11 female and 12 male infants). There was a wide range of gestational ages and birth weights for infants where reorientation of care was recorded (from 22 + 1–23 + 6 weeks and 388–750 grams at birth). Timing of reorientation was variable, in some cases occurring rapidly after admission to NICU (within 6 hours of age) and in others, several months into the neonatal journey (>170 days of life). Similarly, the context for reorientation was variable over time with extreme prematurity being cited as the concomitant cause of death for all infants who died within the first 48 hours of life, whereas reorientation of care was recorded alongside sepsis, necrotising enterocolitis and chronic lung disease in cases where the infant died later.

### 3.4 Extent of stabilisation/resuscitation at birth

**Figure 3:**
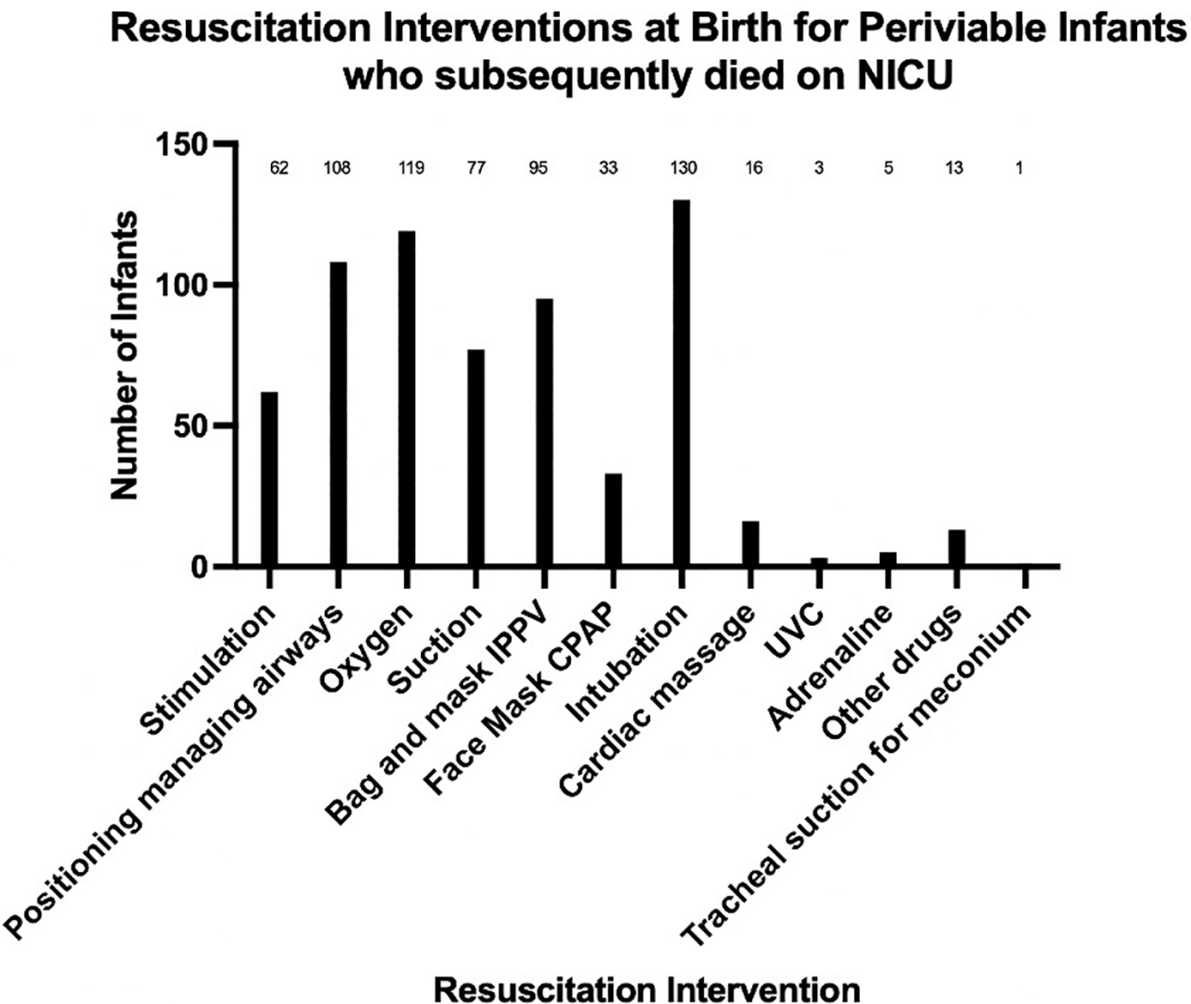
Resuscitation interventions at birth for periviable infants receiving active-focused care who subsequently died in neonatal intensive care units. (NICU, neonatal intensive care unit; IPPV, intermittent positive pressure ventilation; CPAP, continuous positive airway pressure; UVC, umbilical venous catheter).

Data were gathered about the extent of stabilisation and resuscitation interventions provided at birth for these periviable infants (Figure 3). There were eleven infants where there were no details recorded about their stabilisation at birth. For the remaining 132 infants, the majority had received stimulation, positioning managing airway support, supplemental oxygen, suction and intubation at birth. In 33 cases the infant had been supported with face mask continuous positive airways pressure (CPAP) during the stabilisation process. There were sixteen infants where chest compressions had been performed, three where insertion of an umbilical venous catheter in the delivery room was recorded and five where delivery room adrenaline had been administered.

For the 16 infants who received chest compressions, the gestational age range varied from 22 + 0 to 23 + 6 weeks with birth weights from 371 to 720 grams and were spread across the 5-year period (2018–2022). Seven of these infants subsequently died in the first 24 hours (44%) following admission to NICU and a further four infants who had received CPR at birth died within the first week of life (69%). The five infants who received delivery room adrenaline and subsequently died, had variable timing of death from 0 to 74 days after birth.

## 4 Discussion

The data demonstrate that for periviable infants who died following admission to NICU, the majority died within the first seven days after birth (65% of those born at 22 weeks and 52% of those born at 23 weeks). For those infants born at 22 weeks who died on NICU, 89% died within the first 14 days after birth. This indicates that, in most cases, where active, survival-focused care has been initiated after birth, the response of the infant and their individual physiology to this intensive care will become clear over a relatively short-term timeframe after admission and direction of care can then be tailored to that individual infant accordingly.

This concept, that of initiating intensive care and subsequent assessment of the infant's response with a view to determining if continuation of intensive care or reorientation to comfort care is warranted, may be referred to as a “Trial of Life” or “Trial of Therapy” (20). This phrasing, with the legal, potentially combative, associations of the word “trial”, can seem misplaced when attempting to provide individualised care which is sensitive and supportive to an extremely premature infant and their family. Some professionals may refer to the same concept as delayed withholding or withdrawal of intensive care (21). Again, the language used here may be perceived as having negative connotations. Whilst there is a plethora of research outlining the ethical equivalence of withholding vs. withdrawal of treatments (22), there persists a discordance with how these are viewed by professionals in practice. The lived reality of not starting an intervention compared to removing an intervention once started can feel different, regardless of what the academic ethical literature purports (23). The basic ‘Trial of Therapy’ concept, however, would appear to provide the professional and parents with a window of time to assess the individual infant's physiological reaction to the process of birth and initial stabilisation. This can allow for more individualised risk assessment of mortality and morbidity. As the data show, in most cases where the periviable infant died following admission to NICU this was within the first 14 days of life. In cases where pre-delivery discussions between parent and professional have concluded that it would be reasonable to provide active, survival-focused care after delivery, the trial of therapy approach would seem to offer an acceptable balance of time for assessment of the infant and time for the family to spend with their baby, whilst avoiding inflicting an undue burden of harm and distress to the infant. In these situations, high quality palliative care and parallel planning is vital. Palliative care is not synonymous with end-of-life care. It is instead a dynamic process of integrating active treatment with symptom management, holistic support and an acknowledgement of prognostic uncertainty (24). Parallel planning acknowledges the inherent uncertainty in cases of periviable birth and is an approach that allows for creation of individualised management plans that can flex to the reality of changing circumstances (25). The process of parallel planning can be commenced during the pre-birth discussions between parents and professionals and should incorporate not simply the clinical aspects of care (for example, extent of airway management, etc) but rather a holistic approach including information about options for parental contact with baby after birth, memory making options, inclusion of the wider family and religious or spiritual practices (26). Implementing this palliative approach can be complex for the perinatal team. In the face of significant uncertainty, a natural human response can be to attempt to control the situation by favouring structure and a defined plan (27). In a busy clinical environment with multiple perinatal professionals attempting to provide care to numerous patients and their families, successful implementation of a parallel plan across a changing clinical circumstance can seem daunting. Senior medical, midwifery and nursing input is necessary throughout to provide the experience and communication skill set needed to responsively recognise when to move between the different facets of the parallel plan (28, 29).

### 4.1 Reorientation of care

The data show that of the 143 periviable infants included in this study who died after admission to NICU, only 23 infants had reorientation of care (RoC) recorded as a factor at the time of their death (16%). The proportion of infants with RoC recorded was similar across the various timing of death cohorts. Given that periviability is a condition recognised to have high levels of prognostic uncertainty and high rates of mortality and morbidity, it is acknowledged within the recently released BAPM Perinatal Palliative Care framework as a condition where palliative care would be appropriate (24). As outlined earlier, having a palliative condition does not equate to instigation of end-of-life care. However, it may be expected that for periviable infants who died whilst receiving care within NICU, that rates of RoC would be higher than that found in our dataset of merely 23 cases (16%). This raises the question of whether this represents a reluctance to reorientate care for these infants once intensive care has been started, or a hesitancy to record this within the discharge summary. Given the increasing numbers of periviable infants receiving active-survival focused care it will be essential going forward to have robust processes in place to interrogate approaches, management, and outcomes for these infants in order to allow clinicians to better prepare parents and improve management for future infants. This is only possible if data entry into record systems accurately reflects the care provided, including accurate representation of the circumstances of death, of which, reorientation of care may be a relevant aspect. From the available extracted data for infants where RoC was not recorded at the time of death, it was not possible to ascertain if reorientation had been deemed inappropriate by the treating clinical team and/or had never been discussed with the parents or whether RoC had been discussed with and declined by parents. As previously stated, the ethics of withholding and withdrawing a medical intervention are academically generally viewed to be ethically equivalent. However, research shows that for healthcare professionals, particularly those with less experience, the perception can be that these are ethically different (30). Given that Badger.Net discharge summaries are more usually completed by the more junior members of the neonatal team, the low rates of RoC present in our dataset may represent lack of recognition that this is a pertinent piece of information and/or may indicate an element of discomfort or ethical distress. Moral distress within neonatal physicians is an under-researched area and could warrant further exploration, particularly in relation to periviability (31).

### 4.2 Limitations

One limitation of this study is the reliance on the quality and accuracy of data entry into the Badger.Net system by the treating clinicians at the time of the infant's admission and throughout their neonatal journey. Badger.Net is a well-established electronic records system utilised across neonatal units around the United Kingdom and data recorded within the Badger. Net system is used for national quality and research initiatives such as the National Neonatal Audit Programme (NNAP) (32) and the National Neonatal Research Database (NNRD) (33). For this study, utilisation of data from the NW Network Badger. Net systems allowed anonymised collation of data from across the region, representing a larger cohort of periviable infants, rather than a single centre retrospective review.

A further limitation was that whilst Badger. Net has a dedicated section for recording the causes of death as they appear on the medical certificate of cause of death (MCCD), it is not mandatory for the MCCD information to be input into these Badger.Net data fields. Therefore, completion of these data fields is variable, depending on the individual unit practices for completing the Badger. Net discharge letter for babies who have died. For this study, we were therefore, unable to confirm the official diagnoses recorded on the issued MCCD. Instead, data were collated from Badger.Net for cause of death (if recorded) and principal diagnoses at time of death.

## 5 Conclusion

There are increasing numbers of periviable infants being admitted to neonatal intensive care units to receive active, survival-focused care. Periviable birth confers a high risk of mortality and morbidity to the infant. Our data show that the highest risk period for death within NICU for periviable infants was within the first seven days after birth, with continued high risk over the second week of life. Following day 14 of life, the risk of death decreased across both the 22- and 23-week cohorts.

Despite evidence-based risk assessment tools being present in national frameworks for practice, it remains exceedingly difficult for healthcare professionals to be able to accurately predict pre-birth which infants will survive and which will sadly die despite active, survival-focused management. Provision of intensive care is invasive and can cause harm to the infant and emotional and moral distress for the family and healthcare professionals. This data show that in the majority of cases where survival-focused care has been attempted and the infant subsequently died, the response to this active management emerged more clearly over the first 14 days of the admission. This may support the argument that a trial of therapy approach, with robust parallel planning integrated throughout, is an ethically justifiable course of action, balancing the need to avoid prolonged suffering secondary to provision of intensive care, with the desire to provide the infant with a chance at life.

## Data Availability

The data analyzed in this study is subject to the following licenses/restrictions: secondary analysis of North West Neonatal Operational Delivery Network routinely collected admission data. Requests to access these datasets should be directed to jennifer.peterson@hotmail.co.uk.
